# Editorial

**Published:** 1872-05-01

**Authors:** 


### MEDICAL EXAMINER.

*A Semi-Monthly Journal of Medical Sciences.*

EDITED BY

N. S. DAVIS, M. D., AND F. H. DAVIS, M. D.

Chicago, May 1st, 1872.

### EDITORIAL.

AMERICAN MEDICAL ASSOCIATION.—This truly national organization held its annual meeting in Philadelphia, commencing on Tuesday, May 7th, 1872; and closing at noon of the Friday following. It was perhaps the largest, and certainly one of the most cordial and harmonious meetings that have been held since the first preliminary meeting in 1846. We were assured that over 700 delegates and members were present, representing very generally all parts of the Union, from the Atlantic to the Pacific, and from the great Lakes to the Gulf of Mexico. The grand Hall of the Horticultural building on Broad street, was well filled with members and the galleries with spectators. Good section rooms had been provided in the same building, and every arrangement made for the comfort and convenience of members. The Hall, however, like all Music Halls, proved a very bad one to hear or transact business in, and on the morning of the second day the general meeting of the Association was moved into a Church near at hand, which proved so much more comfortable that it continued to be occupied until the close of the session. During the first morning session a large number of reports and papers were announced and referred to the appropriate sections, and the president Dr. D. W. Yandell, of Kentucky, delivered his annual address. The address though lengthy, was listened to with fixed attention and unabated interest to its close. The historical reminiscences forming the introductory part were not only appropriate, but elegant and touching. The main theme of the speaker, was the same, however, as that which had occupied the attention of most of his predecessors, namely, medical education. He justly criticised the extravagant expressions of several of our most eminent men, such as Chapman, Gross, Baldwin and others in reference to the corrupt and degraded condition of medical education in this country, showing that instead of deteriorating either in education or social position, our profession was constantly advancing in full accord with the advancement of science generally; and that it had never held a higher or more influential position in the world than at the present time. Had the speaker left the subject at this point of its discussion, we should have heard nothing causing a feeling of regret. But in the course of his argument there were so many expressions and illustrations that seemed to apologize for, if not positively commend, downright ignorance on the part of medical men, as to materially lessen the pleasure with which we listened to the whole. In all our discussions on this important subject during the last quarter of a century, the disputants have constantly exhibited a tendency to array themselves at opposite extremes. One class constantly represent the profession as steadily deteriorating both in education and social position, and point to the longer periods of professional study, closer adherence to a knowledge of the classics, etc., in Europe, as the proper remedies to arrest the supposed downward tendency. The other class not only deny that any deterioration has taken place, and claim that the education of the profession in this country, in the departments of strict practical importance, is equal to that of any of the countries of Europe; but also that it is advancing as fast as the demands of the people require, and even ridicule the idea of copying after the more extended curriculi of the European schools. They ask with an air of triumph, what has logic or philosophy to do with skill in amputating a limb? And what do the people care for Greek or Latin or even for English orthography when they desire to be cured of a pneumonia or a fever? It has appeared to us, that both these classes are occupying untenable grounds, and both do much to retard genuine and much needed improvements. For instance we can find, in the history of the past, no evidence that the profession in this country ever occupied a higher position either educationally or socially than it does now. We can find much, however, that indicates important advancement in both. But this does not prove that our system of medical education is all right ; or that gross and shameful ignorance of even common branches of an ordinary district school education does not exist with many members of the profession who hold diplomas granted by the medical colleges. It is true, that a man of good natural endowments may acquire skill as an operative surgeon, or become successful as a routine practitioner in the treatment of diseases, while extremely ignorant of either orthography, physical science or classics. And if cutting off limbs or looking at the tongue and writing prescriptions, constituted the whole duty of a physician, we might possibly frame a plausible excuse for tolerating gross ignorance of all general sciences on the part of medical men. It must be remembered, however, that it is as much the duty of the physician to investigate the causes of disease; the means for preventing or obviating the effects of those causes; and thereby contribute to the promotion of the public health, as it is to prescribe for the sick. It is certainly as much his *duty* to record the results of his observations and experience, and contribute them to the general stock of knowledge, as it is his privilege to avail himself of the observation and experience of others for his own benefit and that of his circle of patients. But how is he to discharge these important duties, while ignorant alike of general science and of his own mother tongue.

If those who take upon themselves the task of discussing the subject of medical education would omit their dismal croakings about the positive deterioration of the profession, on the one hand ; and the seeming apologies for stupid ignorance on the other ; and unite in an honest and intelligent effort, not to introduce the complicated and cumbersome educational system of Europe, unsuited as it certainly is, both to the genius of our people and the real wants of our profession, but to improve our standard of preliminary education, properly grade or systemize the college courses of instruction, and restrict the colleges to a rivalry as to which should afford the most complete facilities for medical instruction in every department instead of a struggle to see which can confer the greatest number of Diplomas by making the terms on which they are granted as easy as possible, we should make far greater progress in the work of genuine improvement. As we shall give a full abstract of the proceedings in the next number of the EXAMINER, we will only add here that the president discharged his duties with efficiency, business was transacted with order and decorum, and the session closed with more cordiality of feeling among the members from all parts of the Union than for many years before. Dr. Thomas M. Logan, of Sacramento, California, was chosen president for the ensuing year, and St. Louis was selected for the next annual meeting.

ASSOCIATION OF AMERICAN MEDICAL EDITORS.—A full record of the proceedings of the recent annual meeting of this organization is given in another part of this number of the EXAMINER. The address of the president, Dr. B. F. Dawson, was interesting as affording a glance at the first dawnings of medical knowledge. We shall give a part, or if not found too lengthy, the whole in a future number of the EXAMINER.

CORRECTION.—We learn that a mistake was made in the mailing of the last number of the EXAMINER, by which a few copies of the number for March 15th, was sent instead of April 15th. If any of our subscribers do not receive the proper number, and they inform us the error shall be corrected.

PHAGEDÆNA OF THE PENIS TREATED BY OPIUM.—Mr. Pollock, of St. George's Hospital, treated successfully a patient suffering with the above disease, with tincture of opium. He gave fifteen drops every four hours for two days, and then increased it to twenty drops, with a half drachm of the compound spirit of ammonia. A weak solution of carbolic acid was applied locally on lint. A speedy improvement was observed, and cicatrization was almost complete in a fortnight.—*London Pract.*

